# Two rare cases of oral metastasis arising from lung adenocarcinoma and esophageal carcinoma

**DOI:** 10.4317/jced.57125

**Published:** 2020-10-01

**Authors:** Breno-Amaral Rocha, Lívia-Maris-Ribeiro Paranaíba, Ciro-Dantas Dantas, Maria-Goretti-Freire de Carvalho, Mário-Rodrigues de Melo-Filho, Lucianne-Maia-Costa Lima, Giovanna-Ribeiro Souto, Martinho-Campolina-Rebello Horta

**Affiliations:** 1 Graduate Program in Dentistry, School of Dentistry, Pontifícia Universidade Católica de Minas Gerais (PUC Minas), Belo Horizonte, Minas Gerais, Brazil; 2 Department of Pathology and Parasitology, Institute of Biomedical Sciences, Federal University of Alfenas, Alfenas, MG, Brazil; 3 Department of Oral Diagnosis, School of Dentistry, State University of Campinas, Sao Paulo, Brazil; 4 Full professor, Pathology Department, Potiguar University, Natal, Brazil; 5 Dental School, University of Montes Claros, Montes Claros, Minas Gerais, Brazil; 6 Radiotherapy Service, Santa Casa Hospital, Montes Claros, MG, Brazil

## Abstract

Metastasis to the oral cavity are rare, representing only 1% of all oral malignancies, and originate from various sites such as the breast, prostate, lung and kidney. Clinically, they can simulate reactive and inflammatory lesions common in the oral cavity, and the clinical and microscopic diagnosis of these metastasis is a challenge. In this article, we report two new cases of esophageal and lung metastasis to oral tissues, highlighting their clinical characteristics and the process of diagnostic elucidation. We emphasize the importance for clinicians to consider the possibility of metastatic lesions in the oral cavity in patients previously diagnosed with malignant lesions in distant tissues and organs.

** Key words:**Diagnosis, esophageal squamous cell carcinoma, adenocarcinoma of lung, oral cavity, metastasis.

## Introduction

Metastatic tumors of the oral cavity are uncommon, accounting for only 1% of all oral malignancies (1,2). These tumors, however, are of great clinical significance, since they may represent the first evidence of the dissemination of another (previously unknown) tumor from its primary site, or the first evidence of dissemination of a known tumor from its primary site (3). These lesions may occur in the oral soft tissues, in the jawbones or in both osseous and soft tissue. When it occurs in the jawbones, the mandible is the most common location, with the molar area being the most frequently involved site. When it affects the oral soft tissues, the attached gingiva is the most affected site (4). Any malignant tumor can metastasize to the oral cavity. Such lesions are most commonly found in males, primarily with lung, followed by prostate, kidney, bone and adrenal malignancies, and in females with breast followed by genital and thyroid malignancies (4,5). There are few case reports of metastasis to the oral cavity from relatively uncommon sites such as esophagus and liver (2,6).

Metastatic solid tumors to the oral cavity are highly indicative of a widespread disease process and can obviously modify the prognosis and treatment strategy. In addition, this condition is associated with poor survival rates, with survival time after oral metastasis diagnosis at 3.7 to 8.25 months (5,7,8). Most patients who present with a metastatic lesion in the oral cavity have already been diagnosed with primary tumors; however, in 23%–30% of cases, oral metastasis is the first manifestation of an undiscovered primary malignancy (4,7,8).

Clinically, the metastatic lesions in the oral cavity mimic both benign and malignant lesions, making the diagnosis challenging and interesting. Benign or inflammatory lesions, which enter into the differential diagnoses, include pyogenic granuloma, peripheral giant cell granuloma, peripheral ossify fibroma, and vascular anomaly. On the other hand, the differential diagnoses for malignant lesions include squamous cell carcinoma, lymphoma, soft tissue sarcoma and salivary gland carcinoma (1,8).

In this article, we report two new cases of metastatic tumor of oral soft tissue and their clinical features and management, highlighting the importance to the clinicians of the possibility of metastatic lesion in the oral cavity in patients previously diagnosed with malignant lesions in distant sites.

## Case Report

-Case 1

**Figure 1:**
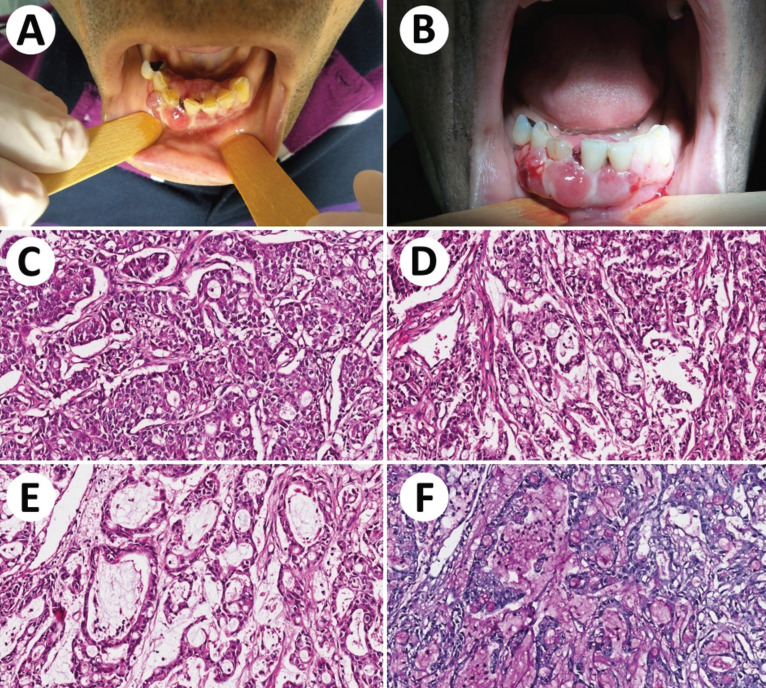
Metastasis of adenocarcinoma from lung to mandible. A: Clinical aspect of the lesion. B: Multinodular enlargements of mandible. C, D and E: Microscopic aspects of the lesion showing an adenocarcinoma with prominent duct-forming structures and large amounts of mucin through the epithelial proliferations. F: PAS stain after diastase digestion highlighted the mucin.

**Figure 2:**
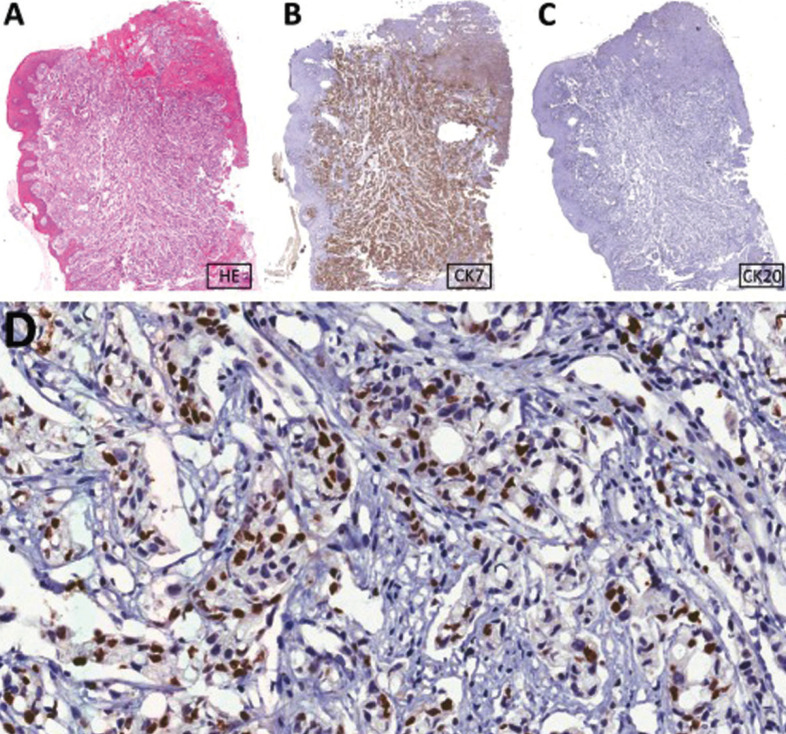
Immunohistochemical features of the lesion. A: HE stain in low magnification. B: Positivity for CK-7. C: Negativity for CK-20. D: Thyroid transcription factor-1 (TTF1) positivity in most of the nuclei of the neoplastic cells.

In February 2015, a 55-year-old male was referred to the Oncologic Dentistry Service with the chief complaint of gingival bleeding and dental mobility. He reported that this complaint had begun five months previously with the appearance of a “lump” in the gingiva. The patient denied any odontogenic symptoms prior to the appearance of the gingival bleeding and dental mobility. The patient reported being an ex-smoker and ex-alcoholic. The patient’s past medical history was significant for adenocarcinoma of the lung (cT4cN2Mx – IIIB). The tumor was unresecTable by invasion of the pulmonary artery, and therefore the treatment plan was radiotherapy and chemotherapy. The physical examination revealed a mobile nodule approximately 1 cm in greatest diameter, subcutaneously, in the right parotid region. Intraoral examination showed an exophytic tissue mass, with a smooth and lobulated surface, firm consistency, and a reddish-pink color located in the gingiva around teeth 32, 31, 41, 42, 43 and 44. The tumor mass measured 6.0 x 3.0 cm. The teeth presented accumulation of biofilm with gingival bleeding to the touch (Fig. 1A,B). Teeth 41 and 42 showed grade 3 mobility. The imaging exams highlighted a radiolucent lytic lesion around teeth 41 and 42. In view of the previous oncological diagnosis and the characteristics of the lesion, the hypothesis of a diagnosis of oral metastasis was considered. The lesion was biopsied and the histology (Fig. 1C-F) and immunohistochemistry analysis of the mass revealed the lesion to be an adenocarcinoma of the lung (Fig. 2A-D) (Table 1). The patient progressed with disease progression and respiratory failure, and one month later died.

**Table 1:**
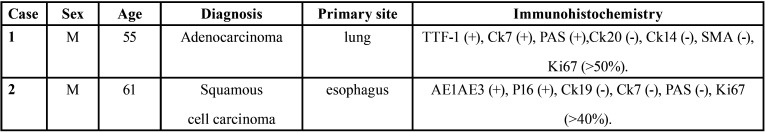
Clinical data and results of immunohistochemical analyses.

-Case 2

**Figure 3:**
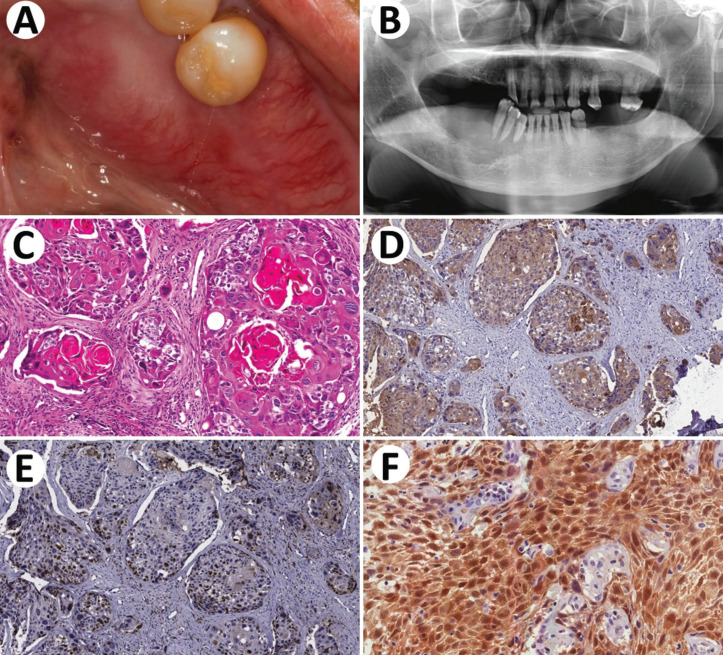
Metastasis of esophageal carcinoma to mandible. A: Clinical aspect of the volumetric increase of the mandible on the right. B: Osteolytic lesion with poorly defined margins involving the right side of the anterior mandible. C: Microscopical aspects include epithelial malignant proliferations with variable amounts of keratin, high atypia and pleomorphism cellular pleomorphism. D, E and F: Immunohistochemical features of the lesion include positivity for pan-Cytokeratin, AE1AE3, high number of Ki67-positive nuclei and high number of p16-positive nuclei, respectively.

In April 2016, a 61-year-old male was referred to the Oncologic Dentistry Service for evaluation of a gingival lesion to be clarified. The patient complained of dental mobility and gingival swelling for approximately the past 15 days. He had local discomfort and hypoesthesia on the lower lip on the right. The patient reported being a former smoker and ex-alcoholic. The patient’s past medical history revealed an oropharyngeal cancer treated in 2013 and a squamous cell carcinoma of the esophagus (T3NXM1 - IV) with cerebral metastasis in 2016. He was submitted to radiotherapy directed to the region of supraclavicular fossas, cervico-thoracic and esophageal concomitant to chemotherapy (Al-Sarraf Protocol). He was also submitted to stereotactic cranial radiosurgery covering two metastatic targets. Were administered single doses of 1.500 cGy to the right subcortical parietal lesion and 800 cGy to the right fronto-parietal region near the convexity, respectively. Intraoral examination showed a firm consistency swelling overlapped by mucosa with telangiectasias, located in the region of teeth 43, 44 and 45, extending to the posterior region of the mandible. The lesion extended to the buccal and lingual surfaces of the abovementioned teeth, measuring approximately 5.0 x 2.0 cm (Fig. 3A). Teeth 43, 44 and 45 showed marked mobility. On the panoramic radiograph, there was a radiolucent lesion with irregular “moth-eaten” borders, involving the roots of teeth 43, 44 and 45, extending to the posterior region of the mandible on the right (Fig. 3B). In view of the previous oncological diagnosis and the characteristics of the lesion, the diagnostic hypothesis of oral esophageal metastasis was raised. The lesion was biopsied and histologic and immunohistochemistry examination showed a moderately differentiated squamous cell carcinoma confirming the diagnostic hypothesis (Fig. 3C-F) (Table 1). The patient progressed with disease progression and one month later died.

## Discussion

Invasion and metastasis are the most important causes of cancer-related morbidity and mortality and are the biological hallmarks of malignant tumors. Although millions of cells are released by the tumor into the circulation, very few of these cells are able to complete the metastatic process (5,6). Metastasis to the oral cavity is extremely rare and is associated with advanced stage, with a poor prognosis and short survival (1,4,6). However, because the maxillary bone is not routinely examined at necropsy, the true frequency may be higher (9). In the literature, there are more cases of metastasis to the jawbones than to oral soft tissues (1,10). However, as in the majority of the cases both the bone and the mucosa are affected, the first metastatic site may not always be apparent (11). The clinical presentation of oral metastasis may vary from local swelling or pain to paresthesia and numbness (2,11). Here we describe two cases of metastasis to the oral soft tissue (cavity) originating from the lung and esophagus presenting advanced stage and evolution to death within one month after the diagnosis of metastasis in the mouth.

Lung cancer, one of the most prevalent conditions in the world, is the most frequent cause of cancer-related death worldwide, and smoking is the predominant risk factor (11-13). Moreover, the lung is the most common source for metastasis to the oral soft tissues in men, and the attached gingiva is the most common affected site followed by the tongue (1,6,8). Allon *et al.* showed that the gingiva that is prone to inflammation may serve as a pre-metastatic niche for the attraction of circulating malignant cells (5). Histologically, the primary foci of lung cancer are usually carcinomas, and among them adenocarcinomas are more common (2). In the 49 cases reviewed by Irani *et al.*, the range of age of the patients at diagnosis with oral metastasis was 45 to 78 years, the time intervals from lung cancer to diagnosis of oral metastasis was one month to five years, the range of time duration of development of a metastatic lesion was one week to three years, and the time intervals from diagnosis to death were two weeks to 26 months (2). The age of the patient in Case 1 was 55 years and the initial detection time of the primary lung carcinoma and diagnosis of the oral metastasis was one month as was the death after diagnosis of metastasis in the mouth. The metastatic lesion appeared one month after the diagnosis of the primary tumor. Gingival bleeding and dental mobility presented by the patient are common clinical presentation of oral metastasis (2,11).

Esophageal cancer is one of most aggressive cancers. Expressive part of the cases spread to the head and neck region and are designated as stage IV (14). Most cases affect men between the ages of 30 and 69 years, with the lower gingiva, the tongue and the floor of the mouth being the most affected sites. Adenocarcinoma represents the most prevalent histological type (2,5,12). The time intervals from esophageal cancer detection to diagnosis of oral metastasis is between one month and seven years, the range of time duration for the development of metastatic oral lesion is one to four months and the time intervals from diagnosis to death is one to four months (2). The age of our patient in Case 2 was 61 years, the initial detection time of the primary esophageal carcinoma and diagnosis of the oral metastasis was four months and the death after diagnosis of metastasis in the mouth was one month. The patient’s smoking history, along with advancing age would be major risk factors for the development of carcinoma (12,13). It is important to note that rapid swelling, pain and paresthesia can be cardinal symptoms of oral metastasis (2,4), as in our case where the patient also presented with hypoesthesia on the lower lip on the right. Advances in the treatment of cerebral metastasis through stereotactic radiosurgery, in addition to new classes of chemotherapy, target drugs and immunotherapy, are responsible for an average increase of two to three times in the survival of patients with brain metastasis during the past decade. The greater survival length achieved by patients with metastatic disease may be contributing to new patterns of recurrence, as observed in the case described (15). Our case, although unusual, corroborates the literature regarding the aggressive nature of esophageal cancer.

Clinically the two lesions presented evidence of an increase in volume which is well reported in the literature as a relatively common pattern in oral metastasis. However, such characteristics should alert dentists, as well as general physicians, to the possibility of there being a metastatic oral disease, especially in patients with known malignant disease. Thus, metastatic tumors in the oral cavity are always challenging to diagnose and these lesions can mimic inflammatory and reactive lesions, which are common in the oral cavity, and other tumors should be considered in the differential diagnosis. It should be noted that the microscopic and immunohistochemical patterns of oral lesions are important for proper diagnosis.

## References

[1] Hirshberg A, Berger R, Allon I, Kaplan I (2014). Metastatic tumors to the jaws and mouth. Head Neck Pathol.

[2] Irani S (2017). Metastasis to the Jawbones: A review of 453 cases. J Int Soc Prev Community Dent.

[3] Bodner L, Sion-Vardy N, Geffen DB, Nash M (2006). Metastatic tumors of the jaws: a report of eight new cases. Med Oral Patol Oral Cir Bucal.

[4] Hirshberg A, Shnaiderman-Shapiro A, Kaplan I, Berger R (2008). Metastatic tumours to the oral cavity - pathogenesis and analysis of 673 cases. Oral Oncol.

[5] Allon I, Pessing A, Kaplan I, Allon DM, Hirshberg A (2014). Metastatic tumors to the gingiva and the presence of teeth as a contributing factor: a literature analysis. J Periodontol.

[6] Rao RS, Patil S, Sanketh Ds, Amrutha N (2014). Metastatic tumors of the oral cavity. J Contemp Dent Pract.

[7] Murillo J, Bagan JV, Hens E, Diaz JM, Leopoldo M (2013). Tumors metastasizing to the oral cavity: a study of 16 cases. J Oral Maxillofac Surg.

[8] Owosho AA, Xu B, Kadempour A, Yom SK, Randazzo J, Ghossein RA (2016). Metastatic solid tumors to the jaw and oral soft tissue: A retrospective clinical analysis of 44 patients from a single institution. J Craniomaxillofac Surg.

[9] Meyer I, Shklar G (1965). Malignant tumors metastatic to mouth and jaws. Oral Surg Oral Med Oral Pathol Oral Radiol Endod.

[10] Nawale KK, Vyas M, Kane S, Patil A (2016). Metastatic tumors in the jaw bones: A retrospective clinicopathological study of 12 cases at Tertiary Cancer Center. J Oral Maxillofac Pathol.

[11] Ito H, Onizawa K, Satoh H (2017). Non-small-cell lung cancer metastasis to the oral cavity: A case report. Mol Clin Oncol.

[12] Lawes KP, Danford M, Di Palma S (2013). Delayed Metastasis to the Mandible of Esophageal Adenocarcinoma. Head and Neck Pathol.

[13] Gultekin SE, Senguven B, Gonul II, Okur B, Buettner R (2016). Unusual Presentation of an Adenocarcinoma of the Lung Metastasizing to the Mandible, Including Molecular Analysis and a Review of the Literature. J Oral Maxillofac Surg.

[14] Shaheen O, Ghibour A, Alsaid B (2017). Esophageal Cancer Metastasis to Unexpected Sites: A Systematic Review. Gastroenterol Res Pract.

[15] Paixão JV, Silva MLG, Chen MJ, Gondim GRM, Fogarolli RC, Pellizzon C (2017). The shifting Landscape of Overall Survival in Patients with Brain Metastasis in a 10-Year Timeframe. Int J of Radiat Oncol Biol Phys.

